# Nonsurgical Strategies in Patients With NET Liver Metastases: A Protocol of Four Systematic Reviews

**DOI:** 10.2196/resprot.2893

**Published:** 2014-03-07

**Authors:** Perparim Limani, Christoph Tschuor, Laura Gort, Bettina Balmer, Alexander Gu, Christos Ceresa, Dimitri Aristotle Raptis, Mickael Lesurtel, Milo Puhan, Stefan Breitenstein

**Affiliations:** ^1^Division of Visceral and Transplantation SurgeryDepartment of SurgeryUniversity Hospital ZurichZurichSwitzerland; ^2^Institute for Social and Preventive MedicineUniversity of ZurichZurichSwitzerland; ^3^Cantonal Hospital WinterthurDepartment of SurgeryWinterthurSwitzerland

**Keywords:** neuroendocrine tumor; NET; liver resection; adjuvant; neoadjuvant; liver transplantation; primary NET; systematic review

## Abstract

**Background:**

Patients diagnosed with neuroendocrine tumors (NETs) with hepatic metastases generally have a worse prognosis as compared with patients with nonmetastasized NETs. Due to tumor location and distant metastases, a surgical approach is often not possible and nonsurgical therapeutic strategies may apply.

**Objective:**

The aim of these systematic reviews is to evaluate the role of nonsurgical therapy options for patients with nonresectable liver metastases of NETs.

**Methods:**

An objective group of librarians will provide an electronic search strategy to examine the MEDLINE, EMBASE, and The Cochrane Library (Cochrane Database of Systematic Reviews, Database of Abstracts of Reviews of Effects, Cochrane Central Register of Controlled Trials [CENTRAL]) databases. There will be no restriction concerning language and publication date. The qualitative and quantitative synthesis of the systematic review will be conducted with randomized controlled trials (RCT), prospective, and retrospective comparative cohort, and case-control studies. Case series will be collected in a separate database and only used for descriptive purposes.

**Results:**

This study is ongoing and presents a protocol of four systematic reviews to assess the role of nonsurgical treatment options in patients with neuroendocrine liver metastases.

**Conclusions:**

These systematic reviews, performed according to this protocol, will assess the value of noninvasive therapy options for patients with nonresectable liver metastases of NETs in combination with invasive techniques, such as percutaneous liver-directed techniques and local ablation techniques.

**Trial Registration:**

International Prospective Register of Systematic Reviews (PROSPERO): CRD42012002657; http://www.metaxis.com/PROSPERO/full_doc.asp?RecordID=2657 (Archived by WebCite at http://www.webcitation.org/6NDlYi37O); CRD42012002658; http://www.metaxis.com/PROSPERO/full_doc.asp?RecordID=2658 (Archived by WebCite at http://www.webcitation.org/6NDlfWSuD); CRD42012002659; www.metaxis.com/PROSPERO/full_doc.asp?RecordID=2659 (Arichived by Webcite at http://www.webcitation.org/6NDlmWAFM); and CRD42012002660; http://www.metaxis.com/PROSPERO/full_doc.asp?RecordID=2660 (Archived by WebCite at http://www.webcitation.org/6NDmnylzp).

## Introduction

### Neuroendocrine Tumors

Neuroendocrine tumors (NET) arise from neuroendocrine cells and are a heterogeneous group of neoplasms [1-3]. NETs originate from a wide range of anatomic sites, which are mainly located in the gastroenteropancreatic system (60%) and the bronchopulmonary system (>25%) [4,5]. With an incidence of 5.25 per 100,000 each year in the United States, NETs are considered to be rare tumors [4].

Patients with NET liver metastases either complain of abdominal pain due to the mass effect of the tumor or excessive hormone production leading to the carcinoid syndrome, which consists of diarrhea, cutaneous flushing, various hemodynamic alterations, and wheezing [6,7]. Moreover, up to 75% of patients with NETs (including midgut or hindgut origin) present with liver metastasis at the time of diagnosis [8].

A 5-year survival rate of 22% for patients with NET liver metastases has been described [5]. Surgical approach shows a benefit concerning overall survival as compared with the nonsurgical approach; however, curative surgery is only applicable in 10% of the patients [9]. Therefore, noninvasive alternatives, such as local ablation techniques, percutaneous liver-directed techniques (chemoembolization, bland embolization, and selective internal radiation therapy), peptide receptor radionuclide technique, chemotherapy, targeted therapy, and biotherapy are of great importance and their value has to be determined.

### Local Ablative Techniques

Mechanistically, local ablative techniques such as cryotherapy, radiofrequency ablation (RFA), and percutaneous alcohol injection (PEI) rely on the cytotoxic effects of chemicals and induce nonphysiologic temperatures into the hepatic tissue [10,11]. Therefore, tumor location and extension in the liver influences the choice of treatment modality [12].

### Percutaneous Liver-Directed Techniques

Treatment modalities involved in percutaneous liver-directed techniques, include bland embolization (BE), transarterial chemoembolization (TACE), and selective internal radiation therapy (SIRT). The principle of BE consists of inducing regional ischemia to the tumor. In TACE, chemotherapeutic substances are locally applied causing a cytotoxic effect. Due to the local embolization the intratumoral concentration of the cytostatic is as much as 20 times higher using TACE than in systemically applied chemotherapy [13]. In addition, systemic side effects can be minimized and cytotoxicity can be maximized [14]. SIRT uses intracorporal radiation through microspheres made of glass (Thera-Spheres) or of resins (SIR-Spheres). These microspheres are loaded with radioactive Yttrium-90 [2]. By virtue of their size, the microspheres obliterate the vessels and irradiate the tumor with a high radiation dose. The adjacent healthy tissue receives minimal dosage [2]. Unfortunately, these treatment modalities are difficult to compare due to the small number of patients and to heterogeneous inclusion criteria (eg, tumor staging, primary tumor location, etc). [15].

### Peptide Receptor Radionuclide Therapy

Peptide receptor radionuclide therapy (PRRT), a combination of a somatostatin analog with a radioligand, a beta-emitter, is used to detect and treat NETs expressing somatostatin receptors. After systemic injection, the radioligand is internalized into the cells and transported to the lysosomes. The effect on tumor cell proliferation is based on the radiotoxicity of the radionuclide in the deoxyribonucleic acid of the target cell [16]. However, radionuclides should be applied cautiously since side effects, such as bone marrow toxicity, hepatic insufficiency, myelodysplastic syndrome, renal insufficiency, or hematological toxicity might occur. Secondary malignancies such as leukemia are rare, but may also occur [17-19]. Functional imaging (ie, octreoscan or gallium 68 Positron Emission Tomography [PET]) is required to identify the subgroup of patients eligible for PRRT [20,21].

### Systemic Chemotherapy

The role of systemic chemotherapy for NETs with liver metastases has been discussed vigorously. In a prospective study, Moertel et al [22] evaluated streptozotocin as a chemotherapeutic monotherapy and found a significant response; however, the benefit was strongly limited by the renal and hematologic toxicity of streptozotocin, and therefore is not an acceptable treatment option. Studies combining streptozotocin with other agents have been conducted with the aim to decrease the dosage of streptozotocin, and thus reduce its toxicity [23]. For the therapy of metastatic pancreatic NETs, Kouvaraki et al [24] reported that a combined multidrug chemotherapy with fluorouracil, doxorubicin, and streptozotocin showed an acceptable response rate of 39% with responders having both increased progression-free survival and overall survival. However, patients with metastatic midgut NETs treated with this multidrug chemotherapy regimen showed the same survival rates as interferon-based therapy concepts [25]. Since poorly differentiated (G3) gastrointestinal NETs behave like lung neuroendocrine carcinomas (small-cell carcinomas) a platin-based chemotherapy is discussed [12].

### Targeted Therapy

Targeted therapy includes multikinase inhibitors, mammalian target of rapamycin (mTOR) inhibitors, and monoclonal antibodies, which interact with various molecular pathways [26]. Sunitinib, an orally applied multikinase inhibitor, targets vascular endothelial growth factor (VEGF) receptors as well as platelet-derived growth factor receptors, which are often expressed in NETs [27,28]. Everolimus, an mTOR inhibitor, has also shown antitumor activity [29]. Bevacizumab, a monoclonal antibody against VEGF, inhibits angiogenesis in tumors and seems to reduce tumor perfusion [26,30].

### Biotherapy

Biotherapy, using interferon-α and somatostatin analogues such as octreotide and lanreotide, prevents the synthesis of the polypeptide hormones and biogenic amines produced by functional NETs. This provides relief from endocrine symptoms associated with the carcinoid syndrome in 80% of patients [31-34]. The Placebo-Controlled, Double-Blind, Prospective, Randomized Study on the Antiproliferative Efficacy of Octreotide LAR in the Control of Tumor Growth in Patients with Metastatic Neuroendocrine Midgut Tumors reports treatment with octreotide (long-acting release) essentially delays the period to tumor progression in patients with both functionally active and inactive metastatic midgut NETs compared with the placebo-treated group. However, survival analysis could not be performed due to a small number of observed deaths [35].

The aim of these four systematic reviews is to determine evidence for the noninvasive treatment options in terms of symptom relief and tumor control in patients with nonresectable liver metastases of NETs.

## Methods

### Systematic Reviews

The following four systematic reviews dealing with the nonsurgical treatment options of neuroendocrine liver metastases attempt to address the following questions represented in Textbox 1.

Our research results will be reported in accordance with the standards of the Preferred Reporting Items for Systematic reviews and Meta-Analyses (PRISMA)[36].

**Figure 1:**
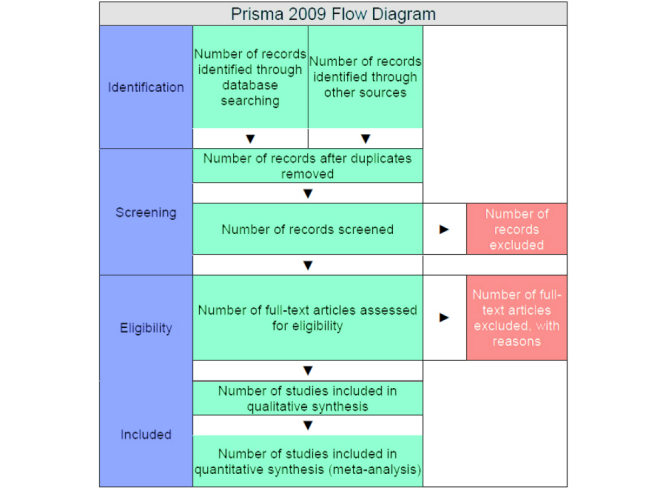
Flow diagram according to PRISMA.

The eligibility criteria for inclusion as well as for exclusion of studies are illustrated in Tables 1-4. Furthermore, the count and reason of exclusion will be revealed in a flow diagram, which will comply with the PRISMA Statement 2009 (Figure 1) [36]. The study types that will be included are randomized controlled trials (RCTs), prospective and retrospective comparative cohort studies, noncomparative cohort studies, case-control-studies, and case series.

These studies will provide the basis for the qualitative synthesis of this systematic review. Single-cohort studies will be collected in a separate database and will only be used for descriptive purposes. No publication date or language restrictions will apply.

**Questions regarding nonsurgical treatment options for neuroendocrine liver metastases.**

1. When should locally ablative techniques (RFA, microwave, and cryotherapy) be used in patients with nonresectable neuroendocrine liver metastases?

- Do local ablation techniques (RFA, microwave, and cryotherapy) improve outcome (progression-free survival, overall survival, and quality of life) in patients with non–resection margin, tumor free (R0)/microscopic tumor lesions (R1) resectable NET liver metastases when compared with nonablative treatments (resection margin, macroscopic lesion [R2] liver resection, percutaneous liver-directed techniques, peptide receptor radionuclide treatment, chemotherapy, targeted therapy, and biotherapy)?
- Which local ablation technique (RFA, microwave, and cryotherapy) achieves the best outcome (progression-free survival, overall survival, and quality of life) in patients with nonresectable NET liver metastases?
- Do local ablation techniques (RFA, microwave, and cryotherapy) in conjunction with a systemic treatment (peptide receptor radionuclide treatment, chemotherapy, targeted therapy, and biotherapy) improve outcome (progression-free survival, overall survival, and quality of life) in patients with nonresectable NET liver metastases as opposed to a systemic treatment alone?
- What is the incidence of tumor dissemination in patients with NET liver metastases undergoing a local ablation technique? Does confirmation occur through imaging/biopsy during the follow-up?

2. When should percutaneous liver-directed techniques be used in patients with nonresectable neuroendocrine liver metastases?

- Do percutaneous liver-directed techniques (bland embolization, chemoembolization, and selective internal radiotherapy) improve outcome (progression-free survival, overall survival, and quality of life) in patients with nonresectable NET liver metastases as opposed to R2 liver resection?
- Which percutaneous liver-directed technique (bland embolization, chemoembolization, and selective internal radiotherapy) achieves the best outcome (progression-free survival, overall survival, and quality of life) in patients with nonresectable NET liver metastases?
- Do percutaneous liver-directed techniques (bland embolization, chemoembolization, selective internal radiotherapy) improve outcome (progression-free survival, overall survival, and quality of life) in patients with nonresectable NET liver metastases in combination with a systemic treatment (peptide receptor radionuclide treatment, chemotherapy, targeted therapy, and biotherapy) when compared with a percutaneous liver-directed technique alone?
- What is the incidence of tumor dissemination in patients with NET liver metastases undergoing a percutaneous liver-directed technique? Does confirmation occur through imaging/biopsy during the follow-up?

3. When should peptide receptor radionuclide therapy be performed in patients with nonresectable neuroendocrine liver metastases?

- Does a peptide receptor radionuclide therapy improve outcome (progression-free survival, overall survival, and quality of life) in patients with nonresectable NET liver metastases when compared with R2 liver resection?
- Does the outcome (progression-free survival, overall survival, and quality of life) for patients with nonresectable NET liver metastases undergoing a peptide receptor radionuclide therapy depend upon the size of liver metastases (>5- vs <5-cm diameter of the largest tumor) or their uptake on a diagnostic scan?
- Does the outcome (progression-free survival, overall survival, and quality of life) of a peptide receptor radionuclide therapy depend upon the percentage of liver volume involvement (eg, <75% vs >75%) for patients with nonresectable NET liver metastases?
- Does the outcome (progression free survival, overall survival, and quality of life) of a peptide receptor radionuclide therapy, for patients with nonresectable NET liver metastases, depend upon the site of the primary tumor?
- Does a peptide receptor radionuclide therapy in combination with percutaneous liver-directed techniques (bland embolization, chemoembolization, and selective internal radiotherapy) and/or local ablation techniques improve outcome (progression-free survival, overall survival, and quality of life) in patients with nonresectable NET liver metastases when compared with peptide receptor radionuclide therapy as a single technique?

4. When should chemotherapy, targeted therapy, or biotherapy be used in patients with nonresectable neuroendocrine liver metastases?

- Does chemotherapy, targeted therapy and biotherapy improve outcome (progression-free survival, overall survival, and quality of life) in patients with nonresectable NET liver metastases as opposed to R2 liver resection?
- Does outcome (progression-free survival, overall survival, and quality of life) of chemotherapy, targeted therapy, and biotherapy in patients with nonresectable NET liver metastases depend upon the size of liver metastases (>5- vs <5-cm diameter of the largest tumor)?
- Does outcome (progression-free survival, overall survival, and quality of life) of chemotherapy, targeted therapy, and biotherapy in patients with nonresectable NET liver metastases depend upon the percentage of liver volume involvement (eg, < 75% vs >75%)?
- Does outcome (progression free survival, overall survival, and quality of life) of chemotherapy, targeted therapy, and biotherapy in patients with nonresectable NET liver metastases depend upon the site of the primary tumor?
- Does chemotherapy, targeted therapy, and biotherapy in combination with percutaneous liver-directed techniques (bland embolization, chemoembolization, and selective internal radiotherapy) and/or local ablation techniques improve outcome (progression-free survival, overall survival, and quality of life) in patients with nonresectable NET liver metastases when compared with chemotherapy, targeted therapy, and biotherapy as a single technique?

**Table 1:** Eligibility criteria for review 1: when should locally ablative techniques be used in patients with unresectable neuroendocrine liver metastases?

Study characteristic	Inclusion criteria	Exclusion criteria
Patients population	Patients with nonresectable NLMs^a^	Children or adolescents (under the age of 18 years)
	Patients that underwent ablation or palliative resection	
Intervention treatment	Palliative surgical resection	
	Ablation (cryo^b^, RFA^c^, LITT^d^, PEI^e^)	
	Systemic treatment (chemotherapy, biotherapy, and targeted therapy)	
Intervention comparison	Surgical resection vs ablation	
	Ablative techniques compared with others	
	Ablation combined with systemic treatment vs ablation only	
Study design	RCTs^f^	Case reports
	Prospective and retrospective single- or multicenter cohort studies	
	Case series	
Reporting		Overall survival not mentioned

^a^Neuroendocrine liver metastases

^b^Cryotherapy

^c^Radiofrequency ablation

^d^Laser induced thermotherapy

^e^Percutaneous alcohol injection

^f^Randomized controlled trials

**Table 2:** Eligibility criteria for review 2: when should percutaneous liver-directed techniques be used in patients with nonresectable neuroendocrine liver metastases?

Study characteristic	Inclusion criteria	Exclusion criteria
Patient population	Patients with nonresectable NET liver metastases	Children or adolescents (under the age of 18 years)
	Patients treated with percutaneous liver directed techniques	
Intervention(s)/ exposure(s)	Percutaneous liver directed techniques (bland embolization, chemoembolization, and selective Internal radiotherapy)	
Comparator(s)/ control	Palliative liver resection	
	Percutaneous liver directed technique with or without systemic treatment	
Study design	RCTs^a^	Case reports
	Prospective and retrospective comparative cohort studies	
	Case-control studies	
	Case series	
Reporting	Primary outcome: overall survival	Studies that do not report the overall survival
	Secondary outcome: progression-free survival, quality of life	

^a^Randomized controlled trials

**Table 3:** Eligibility criteria for review 3: when should peptide receptor radionuclide therapy be performed in patients with nonresectable neuroendocrine liver metastases?

Study characteristic	Inclusion criteria	Exclusion criteria
Patient population	Patients with nonresectable liver metastases treated with peptide receptor radionuclide therapy	Children or adolescents (under the age of 18 years)
Intervention–treatment	Peptide receptor radionuclide therapy	
	Percutaneous liver directed techniques (bland embolization, chemoembolization, and selective internal radiotherapy)	
Intervention–comparison	Palliative resection vs peptide receptor radionuclide therapy	
Study design	RCTs^a^	Case reports
	Prospective and retrospective comparative cohort studies	
	Noncomparative cohort studies	
	Case-control studies	
	Case series	
Reporting		Studies that do not report the overall survival

^a^Randomized controlled trials

**Table 4:** Eligibility criteria for review 4: when should chemotherapy, targeted therapy or biotherapy be used in patients with nonresectable neuroendocrine liver metastases?

Study characteristic	Inclusion criteria	Exclusion criteria
Patient population	Patients with nonresectable NET liver metastases	Children or adolescents (under the age of 18 years)
	Patients that underwent chemotherapy or biotherapy or targeted therapy or palliative liver resection	
Intervention–treatment	Chemotherapy	
	Biotherapy	
	Targeted therapy	
	Chemotherapy or biotherapy or targeted therapy with percutaneous liver-directed techniques (bland embolization, chemoembolization, selective internal radiotherapy)	
	Chemotherapy or biotherapy or targeted therapy with locally ablative techniques	
Intervention–comparison	Chemotherapy or biotherapy or targeted therapy vs palliative resection	
	Chemotherapy or biotherapy or targeted therapy with percutaneous liver-directed techniques vs single therapy	
	Chemotherapy or biotherapy or targeted therapy with locally ablative techniques vs single therapy	
Study design	RCTs^a^	Case reports
	Prospective and retrospective comparative cohort studies	
	Noncomparative cohort studies	
	Case-control studies	
	Case series	
Reporting		Studies that do not report the overall survival

^a^Randomized controlled trials

### Search

The electronic search strategy to scan the databases and detect all relevant articles was developed by the librarians of the Medical Library Careum (University of Zurich, Switzerland). The search will be performed on the MEDLINE, EMBASE, and The Cochrane Library (Cochrane Database of Systematic Reviews, Database of Abstracts of Reviews of Effects, and Cochrane Central Register of Controlled Trials [CENTRAL]) databases. An endnote file, comprising all identified titles and, if accessible, the corresponding abstracts will be prepared for the investigators. Supplementary publications will be found by manual search or by reviewing reference lists. Two independent review group members will peruse titles and/or abstracts from studies, which were identified using the search profile. Afterwards, the entire text of these potentially eligible studies will be re-examined for eligibility. Any uncertainties will be discussed and resolved with a third member. A specially created Web-based, predetermined protocol will be used to extract data from the included studies for the study quality expertise and synthesis of medical findings.

### Data Extraction

The data extraction parameters, include manuscript title, name of journal, first author's name, publication year, total number of patients, number of patients in the chemotherapy/biotherapy/targeted therapy group, number of patients in the nontreatment group, name of used substances, age (mean, standard deviation, median), male to female ratio, progression-free survival, overall survival, quality of life (containing side effects), study design, and targeting objective 1-5. The Grading of Recommendations Assessment, Development and Evaluation (GRADE) will be used to grade the quality (level) of evidence and the strength of recommendations [37].

We will prepare a narrative synthesis of the findings from the included studies. A quantitative synthesis will be used if the included studies are sufficiently homogenous. We anticipate that there will be a limited scope for meta-analysis of a relatively large number of studies because of the range of outcomes measured across the small number of existing trials (such tumors are rare). Nevertheless, where studies have used the same type of intervention and comparator, with the same outcome measure, we will pool the results using a random-effects meta-analysis. We calculate a 95% CI and two-sided *P* values for each outcome.

## Results

This study is ongoing and presents a protocol of four systematic reviews to assess the role of nonsurgical treatment options in patients with neuroendocrine liver metastases. Both noninvasive as well as invasive methods, such as percutaneous liver-directed techniques and local ablation techniques will be investigated.

## Discussion

Several nonsurgical treatment options for neuroendocrine liver metastases have been reported. However, there is a lack of consensual data on the subject. These four systematic reviews described in this protocol aim to clarify the role of nonsurgical therapy modalities in patients with nonresectable NETs liver metastases. The systematic reviews will serve as a basis for developing clinical practice guidelines.

## Abbreviations

BE: bland embolization
CENTRAL: Cochrane Central Register of Controlled Trials
GRADE: The Grading of Recommendations Assessment, Development and Evaluation
mTOR: mammalian target of rapamycin
NET: neuroendocrine tumors
PET: Positron Emission Tomography
PRISMA: Preferred Reporting Items for Systematic Reviews and Meta-Analyses
PRRT: peptide receptor radionuclide therapy
R0, R1, R2: resection margin (R0: tumor free; R1: microscopic lesion; R2: macroscopic lesion)
RCT: randomized controlled trial
RFA: radio frequency ablation
SIRT: selective internal radiation therapy
TACE: transarterial chemoembolization
VEGF: vascular endothelial growth factor (receptor)
